# MAB-Based Online Client Scheduling for Decentralized Federated Learning in the IoT

**DOI:** 10.3390/e27040439

**Published:** 2025-04-18

**Authors:** Zhenning Chen, Xinyu Zhang, Siyang Wang, Youren Wang

**Affiliations:** 1 College of Automation Engineering, Nanjing University of Aeronautics and Astronautics, Nanjing 210016, China; wangyrnuaa@l26.com; 2 School of Computer Science and Technology, Nanjing University of Posts and Telecommunications, Nanjing 210049, China; b22040702@njupt.edu.cn; 3 Jiangsu Key Laboratory of Wireless Communications, Nanjing University of Posts and Telecommunications, Nanjing 210049, China; 2024010101@njupt.edu.cn; 4 Engineering Research Center of Health Service System Based on Ubiquitous Wireless Networks, Ministry of Education, Nanjing University of Posts and Telecommunications, Nanjing 210049, China

**Keywords:** decentralized federated learning, client scheduling, multi-armed bandit

## Abstract

Different from conventional federated learning (FL), which relies on a central server for model aggregation, decentralized FL (DFL) exchanges models among edge servers, thus improving the robustness and scalability. When deploying DFL into the Internet of Things (IoT), limited wireless resources cannot provide simultaneous access to massive devices. One must perform client scheduling to balance the convergence rate and model accuracy. However, the heterogeneity of computing and communication resources across client devices, combined with the time-varying nature of wireless channels, makes it challenging to estimate accurately the delay associated with client participation during the scheduling process. To address this issue, we investigate the client scheduling and resource optimization problem in DFL without prior client information. Specifically, the considered problem is reformulated as a multi-armed bandit (MAB) program, and an online learning algorithm that utilizes contextual multi-arm slot machines for client delay estimation and scheduling is proposed. Through theoretical analysis, this algorithm can achieve asymptotic optimal performance in theory. The experimental results show that the algorithm can make asymptotic optimal client selection decisions, and this method is superior to existing algorithms in reducing the cumulative delay of the system.

## 1. Introduction

The Internet of Things (IoT), driven by the latest advancements in information and communication technologies, connects countless devices to the Internet, enabling seamless connectivity and real-time interaction between people, machines, and objects [1]. Recently, with the development of IoT, a large number of intelligent applications and services based on IoT have begun to emerge; while bringing great convenience to people’s daily work and life, it is also promoting profound changes in the fields of industrial manufacturing, agricultural production, and infrastructure construction [2]. At the same time, the development and widespread use of machine learning [3] makes it possible to mine huge amounts of potential value from the vast amounts of data generated by IoT devices, providing more intelligent solutions to existing and newly developed applications.

However, conventional centralized machine learning methods require user devices to upload their raw data, which may contain sensitive information, thus increasing the risk of privacy leakage [4]. Moreover, uploading large-scale raw data to the central server will consume many network bandwidth resources and cause considerable communication delays. Recently, federated learning (FL) [5] has been introduced as a solution to the aforementioned challenges. In FL, clients do not need to share their local data with the cloud or other clients; instead, they can train models locally and upload the updated model for global aggregation. Therefore, the risk of sensitive data leakage, as well as communication overhead, is significantly reduced. FL has been widely applied in different fields, such as natural language processing [6], intelligent manufacturing [7], intelligent transportation [8], and smart healthcare [9].

Traditional centralized FL systems are susceptible to the single point of failure effects; that is to say, when the central server is broken down, the FL training process cannot continue. Furthermore, the limited wireless resources cannot support ever-increasing user devices to participate in FL training simultaneously, and the scalability of the centralized FL systems is constrained [10]. Thus, decentralized FL (DFL) [11] architecture attracts researchers’ interest, deploying multiple servers and enabling more user devices to collaborate on global model training. DFL can reduce the impact of single server failure on model training and further improve system scalability.

However, the deployment of DFL in IoT still faces challenges [12]. Firstly, different user devices have different computing and communication resources, and we refer to this issue as resource heterogeneity [13]. Secondly, the data collected by different user devices are imbalanced and non-identically and independently distributed (non-i.i.d.), which is referred to as data heterogeneity. Thirdly, because the resource availability of user devices is time-varying, the local training delay and communication delay are difficult to estimate, and the training efficiency of FL cannot be guaranteed [14]. Finally, with the increase of user devices, multiple servers still cannot allow simultaneous access to massive devices due to the limited radio resources. To address these challenges, this work aims to propose a novel client scheduling strategy that selects a portion of clients to participate in the DFL training process in each round. The main contributions of this paper can be summarized as follows:

- This paper considers the client scheduling problem in DFL scenarios. Due to the heterogeneity of local computing and communication resources, as well as the time-varying nature of wireless channels, the total delay of each client in each round cannot be predicted. Thus, we formulate the client scheduling problem as a contextual combinatorial multi-armed bandit (CC-MAB) program [15].
- We propose an online client scheduling algorithm that estimates the delay of clients based on their contextual information during training and continuously updates the estimator according to the actual delay. Through theoretical analysis and algorithm parameter design, this algorithm can achieve asymptotic optimal performance in theory.
- Finally, through extensive experiments, we show that the algorithm can make asymptotically optimal client scheduling decisions, which is superior to existing algorithms in reducing the cumulative delay of the system.

The rest of this work is organized as follows. Section 2 presents the related works. Section 3 and Section 4 introduces the system model and formulates the optimization problem. Section 5 and Section 6 provides the convergence analysis and client scheduling algorithm. Section 7 presents the simulation results, followed by the conclusions in Section 8.

## 2. Related Works

### 2.1. Client Scheduling in Centralized FL

There have been many works studying client scheduling and resource allocation in the traditional centralized FL. The authors in [16] aimed to reduce the communication load on the central server by identifying clients with irrelevant updates and excluding them from model aggregation. In [17], a communication- and computation-efficient client selection method was proposed where the clients with significant local training losses were selected to accelerate model convergence. In [18], the importance of local learning updates was measured based on the gradient differences of local learning updates, and then a client scheduling method was proposed to balance between client channel quality and update importance. A joint optimization method for client selection and wireless resource allocation based on bipartite matching was proposed in [19], which minimized the global training loss function by optimizing the transmission power of client devices and the wireless resource allocation of servers. The authors in [20] modeled the client scheduling problem in wireless FL with unknown client channel states as an MAB program [21] and proposed a solution based on the ϵ-greedy method to balance exploration and exploitation.

There are also some works focusing on optimizing the efficiency of hierarchical FL. For example, the work [22] formulated a joint problem of client scheduling and resource optimization in a hierarchical FL architecture and solved the problem by decomposing the original problem into two sub-problems: resource allocation and edge server cooperation. The authors in [23] simultaneously considered the uncertainty of wireless channels and the weights of client models and transformed the original optimization problem into a mixed integer nonlinear programming problem through theoretical derivation for a solution. The work [24] considered a multi-objective optimization problem under local computing resources and client transmit power constraints and proposed an algorithm based on deep reinforcement learning. In terms of improving system energy efficiency, the work [25] simultaneously considers the local data distribution of clients and the delay caused by model transmission. By jointly optimizing the association strategy between clients and edge servers and resource allocation, the system communication energy consumption is minimized. The work [26] considers the joint optimization of client-server association and client local computing power control under long-term energy consumption constraints to simultaneously minimize global training loss and delay. The authors in [27] studied the client scheduling problem in a hierarchical FL framework and proposed a method based on contextual combined MAB to learn the states of clients who successfully participate in training during the global iteration process, thereby providing appropriate client selection strategies for subsequent training.

### 2.2. Client Scheduling in DFL

Due to limited communication and computing resources, the key to optimizing the DFL performance lies in balancing the number of communication and computing rounds. The authors in [28] proposed a universal DFL framework to achieve a balance between system communication efficiency and global model convergence by performing a certain number of local model updates and model exchanges between nodes in each round of global iteration. Similarly, the authors in [29] considered the resource heterogeneity of different devices and analyzed the impact of local training on the global model convergence. Based on the analysis results, closed-form solutions for local training rounds of different local nodes were obtained. Furthermore, the authors in [30] incorporated the node selection strategy into a regularized multi-objective optimization problem, aiming to maximize system knowledge gain while minimizing energy consumption. In response to the limited node resources in large-scale IoT scenarios, the work [31] proposed a joint optimization method of node scheduling and bandwidth allocation in asynchronous DFL, aiming to minimize the transmission delay of FL models and improve convergence speed.

However, the above literature assumes that there are no model errors or losses during the model propagation process, which is unrealistic, especially in wireless environments. Although the reliability of model transmission can be improved through the use of the transmission control protocol (TCP) [32] and other methods, more additional communication overhead is caused, and the scalability of the system is reduced. To address this issue, the work [33] divided the model parameters into multiple data packets and sent them through the user datagram protocol (UDP) [34]. Then, the weighted matrix of the inter-node model mixture based on the reliability matrix of inter-node communication was optimized.

## 3. System Model

**Figure 1:**
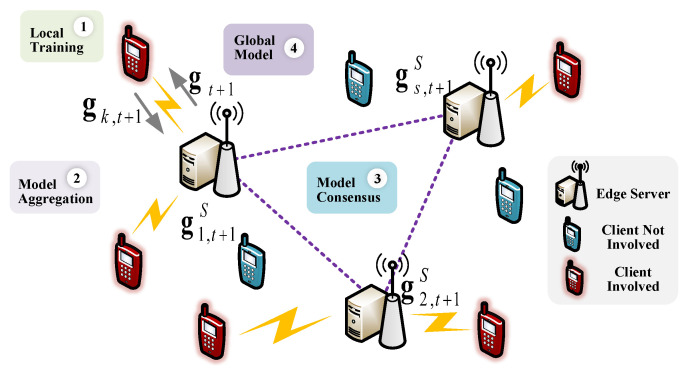
System framework.

We consider an area comprising *S* cellular cells, each served by a base station (BS) located at the center of the cell. Each BS is equipped with an edge server, and the set of these edge servers is denoted by S={1,2,…,S}. There are *K* single-antenna devices (clients), denoted by a set K={1,2,…,K}, that are randomly distributed in the considered area. We assume that each edge server has *N* orthogonal wireless channels. In other words, each server can communicate with up to *N* clients within the communication range of the BS at the same time (See Figure 1). The set of clients in the cell *s* is located is denoted as $K_{s}=\left\{k\in K|k\in \mathrm{cell}\;s\right\}$ with $\left|K_{s}\right|=K_{s}$. In this work, we assume that NS≪K.

All the clients and edge servers in the area are organized to train a shared global model through DFL. The edge servers select the participating clients at the beginning of each training round. We define the binary variable $a_{k}\left(t\right)\in \left\{0,1\right\},\forall k\in K,t\leq T$. Specifically, if device *k* participates in the training of round *t*, $a_{k}\left(t\right)=1$; otherwise, $a_{k}\left(t\right)=0$. We further define the set of clients participating in round *t* as $A\left(t\right)=\{k\in K|a_{k}\left(t\right)=1\}$. Similarly, we denote by $A_{s}\left(t\right)=\left\{k\in K|a_{k}\left(t\right)=1,k\in \mathrm{cell}\;s\right\}$ a set of clients which are associated with edge server *s*. Furthermore, we collect these $a_{k}\left(t\right),\forall k\in K$ into $a\left(t\right)=\{a_{1}\left(t\right),a_{2}\left(t\right),\ldots ,a_{K}\left(t\right)\}$ and denote by $a\overset{\Delta }{\;=}\left\{a(1),a(2),\ldots ,a(T)\right\}$ the client participation metric of all the training rounds.

### 3.1. DFL Process

The goal of DFL is to minimize the weighted global training loss, i.e.,

(1) $$F\left(g\right)=\frac{1}{K}\sum_{k\in K}F_{k}\left(g\right),$$

where g are the global model parameters, and $F_{k}\left(g\right)$ is the local training loss of client *k*, denoted as

(2) $$F_{k}\left(g\right)=\frac{1}{\left|D_{k}\right|}\sum_{d=1}^{D_{k}}f(g,x_{kd},y_{kd}),$$

where $\left|D_{k}\right|$ is the size of the local dataset of client *k*, and $f(g,x_{kd},y_{kd})$ is the local loss function of training data $\left(x_{kd},y_{kd}\right)$.

Each global training round of the DFL involves three phases: (1) local model training, (2) intra-cluster model aggregation, and (3) inter-cluster model aggregation. During the local model training phase, each client updates model parameters based on their local training dataset as

(3) $$g_{k,t+1}=g_{t}-\frac{\lambda }{\left|D_{k}\right|}\sum_{d=1}^{\left|D_{k}\right|}\nabla f(g_{t},x_{kd},y_{kd}),k\in A\left(t+1\right),$$

where $g_{k,t+1}$ denotes the local model of client *k* in round t+1, $g_{t}$ denotes the global model at the end of the previous training round, λ represents the learning rate, and $\nabla f(g_{t},x_{kd},y_{kd})$ is the gradient of the model $g_{t}$ on the training dataset $D_{k}$. In the phase of intra-cluster model aggregation, the clients participating in the training process upload their latest local model parameters to the associated edge servers via cellular communication. Then, each edge server aggregates the received model parameters as follows:

(4) $$g_{s,t+1}^{\left(S\right)}=\frac{\sum_{k\in A_{s}\left(t+1\right)}\left|D_{k}\right|g_{k,t+1}}{\sum_{k\in A_{s}\left(t+1\right)}\left|D_{k}\right|},$$

where $g_{s,t+1}^{\left(S\right)}$ is the model of server *s* after intra-cluster model aggregation in round t+1.

In the phase of inter-cluster model aggregation, each server transmits the updated model to other servers connected to it via high-speed wired links. In this work, we assume that the edge servers are fully connected. Thus, each server aggregates its received models and its models to obtain a global model:

(5) $$g_{t+1}=\frac{\sum_{s\in S}\sum_{k\in A_{s}\left(t+1\right)}\left|D_{k}\right|g_{s,t+1}^{\left(S\right)}}{\sum_{k\in A(t+1)}\left|D_{k}\right|},$$

where $g_{t+1}$ represents the global model obtained during the inter-cluster model aggregation in round t+1. After inter-cluster model aggregation, each server sends the global model back to the associated clients, and the clients then substitute their local models with the global model.

### 3.2. Delay Model

Considering the sufficient computational power of the edge servers, the delay caused by model aggregation at the edge servers can be ignored. Additionally, the transmission delay of model transmission between the edge servers can also be ignored because of the high-speed wired links between these edge servers. Therefore, the total delay of client *k* in training round *t* is as follows:

(6) $$\tau_{k}\left(t\right)=\min\left\{\tau_{k}^{D}\left(t\right)+\tau_{k}^{U}\left(t\right)+\tau_{k}^{\mathrm{LU}}\left(t\right),\tau_{\max}\right\},$$

where $\tau_{k}^{D}\left(t\right)$ denotes the delay of the client downloading the latest global model from the associated server, $\tau_{k}^{U}\left(t\right)$ denotes the delay of the client uploading the local model to the associated server, $\tau_{k}^{\mathrm{LU}}\left(t\right)$ represents the delay caused by local model updates, and $\tau_{\max}$ represents the maximum delay allowed for each training round. Specifically, the delay of client *k* in downloading the global model can be expressed as

(7) $$\tau_{k}^{D}\left(t\right)=\frac{m}{BR_{k}^{D}\left(t\right)},$$

with

(8) $$R_{k}^{D}\left(t\right)=\log_{2}\left[1+\frac{p_{k}^{D}{\left|h_{k}^{D}\left(t\right)\right|}^{2}}{\sigma^{2}}\right],$$

where *m* is the data size of model parameters, *B* is the downlink channel bandwidth, $R_{k}^{D}\left(t\right)$ is the downlink transmission rate, $p_{k}^{D}$ is the downlink transmission power, $h_{k}^{D}\left(t\right)$ is the downlink channel gain, and σ is the noise power spectral density. Similarly, the delay of the client uploading the local model can be expressed as follows:

(9) $$\tau_{k}^{U}\left(t\right)=\frac{m}{BR_{k}^{U}\left(t\right)},$$

with

(10) $$R_{k}^{U}\left(t\right)=\log_{2}\left[1+\frac{p_{k}^{U}{\left|h_{k}^{U}\left(t\right)\right|}^{2}}{\sigma^{2}}\right],$$

where $R_{k}^{U}\left(t\right)$ is the uplink transmission rate, $p_{k}^{U}$ is the uplink transmit power, and $h_{k}^{U}\left(t\right)$ is the uplink channel gain. The local update delay can be expressed as follows:

(11) $$\tau_{k}^{\mathrm{LU}}\left(t\right)=\frac{s_{k}\left|D_{k}\left(t\right)\right|}{\varphi_{k}\eta_{k}\left(t\right)},$$

where $s_{k}$ is the number of CPU rounds required for calculating the unit data volume, $\varphi_{k}$ are the computational resources of client *k*, and $\eta_{k}\left(t\right)$ are the available computational resources of client *k* in round *t*.

## 4. Problem Formulation

In this work, we consider synchronous DFL training, which means that at the intra-cluster aggregation phase of each training round, the server conducts model aggregation only after receiving the models from all clients associated with it. Therefore, the delay of round *t*, denoted by τ(a(t)), is determined by the slowest client, i.e.,

(12) $$\tau^{t}\left(a\left(t\right)\right)=\max_{k\in A(t)}\tau_{k}\left(t\right).$$

Due to the ever-changing wireless channel state and the available resources of client devices, the transmission and computational delay will vary during the training process. To shorten the training time of synchronous DFL, this paper optimizes the client participation scheme in a *T*-round training process to minimize the total delay, i.e.,

(13a) $$\begin{array}{cc} \min_{a} & \sum_{t=1}^{T}\tau^{t}\left(a\left(t\right)\right), \end{array}$$

(13b) $$\begin{array}{cc} \;\;\;\;\;s.t. & a_{k}\left(t\right)\in \left\{0,1\right\},\forall k\in K,t\leq T, \end{array}$$

(13c) $$\begin{array}{cc} & |A_{s}\left(t\right)|\leq N,\forall s\in S,t\leq T, \end{array}$$

where (13c) is the access constraint that limits the maximum number of clients that each edge server can serve in each round. However, due to the uncertainty of wireless channels and client activities, the local processing delay and model transmission delay in (11) are hard to obtain.

Fortunately, we can infer the client delay in each round by observing contextual information. Specifically, the context of client *k* in round *t* is denoted as $x_{k}^{t}\in X$, where $X={\left[0,1\right]}^{D}$. The server estimates the delay of client *k* based on experience $P_{k}$, i.e., ${\hat{\tau }}_{k}\left(t\right)=\Theta_{k}\left(x_{k}^{t},P_{k}\right)$, where $\Theta_{k}\left(\cdot \right)$ is the estimator corresponding to client *k*. Based on the estimated delay of clients in each round, the system selects clients to participate in each training round. Therefore, the problem (13) is re-expressed as follows:

(14) $$\begin{array}{cc} \min_{a}\;\; & \sum_{t=1}^{T}\max_{k\in A(t)}{\hat{\tau }}_{k}\left(t\right)\\ s.t.\;\; & \mathrm{(13b)}\;\mathrm{and}\;\mathrm{(13c)}. \end{array}$$

The key to Problem (14) is how to estimate client delay in each round according to the observed contextual information. In the following, we will introduce the contextual combinatorial multi-armed bandit (CC-MAB) programming [15], based on which a client scheduling algorithm is proposed.

## 5. Algorithm Design

In a CC-MAB problem, the player performs actions by pulling one or more of a set of arms. Every time an action is executed, the player will receive a reward value. Before executing an action, the player first observes the contextual information of each arm, and by recording the reward value obtained from executing the action, the player can obtain the corresponding expected reward of the action in that context. By continuously pulling different arms and recording reward values, players gradually learn the best strategy to maximize the expected reward value. It is worth noting that the action corresponding to the un-pulled arm will not be executed; therefore, no corresponding reward values will be recorded.

In our work, each edge server acts as the player, with its arms being all clients within its coverage area. The action of the server is to select a group of clients for scheduling in each training round, while the action space consists of all possible client combinations, subject to the constraint that each edge server can schedule at most *N* clients simultaneously. The reward obtained by the server in each round is defined as $-\frac{\tau^{t}\left(a\left(t\right)\right)}{\tau_{max}}+1$, where $\tau^{t}\left(a\left(t\right)\right)$ represents the delay in the current round. Therefore, we propose a client scheduling method based on CC-MAB programming, enabling the system to learn the optimal strategy and minimize cumulative delay. The client scheduling process is described as follows.

**Figure 2:**
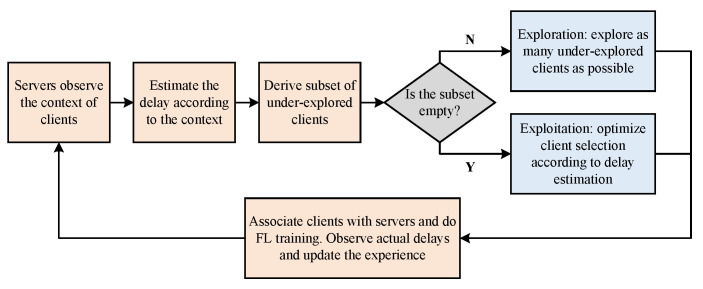
Flow of the proposed algorithm.

Before each training round begins, each edge server observes the context of clients within the cell. Subsequently, the servers estimate the delay of the clients in this round based on their historical experience information and contextual information. Afterward, each edge server determines client selection. After determining the clients participating in the training round, the system organizes the aforementioned clients to perform DFL model training. After the training is completed, each edge server records the actual delay of the clients participating in this training round and adds it to the client’s historical experience information along with the contextual information observed before the next round of training. Figure 2 summarizes the flow of the proposed client scheduling algorithm.

### 5.1. Delay Estimation Based on Contextual Information

**Figure 3:**
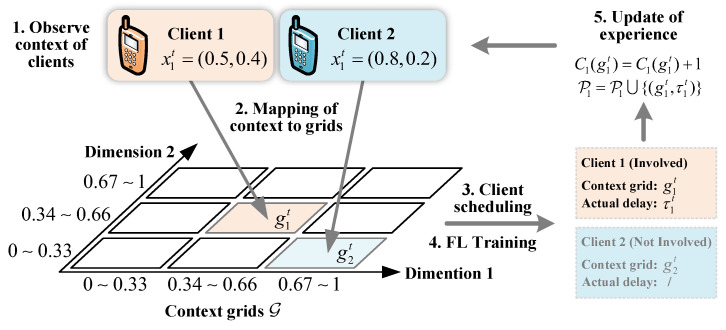
An illustration of the context.

The context of client *k* in round *t* is denoted as $x_{k}^{t}\in X$, where the context space contains *D* dimensions, denoted as $X={\left[0,1\right]}^{D}$. The contextual information considered in this work includes the following: (1) the client’s current device activity I∈I, such as the number of running programs, is reported by the client themselves; (2) the size of the local dataset $D_{k}$ used for training. Due to the continuous values in each dimension of the context space, training an estimator based on every possible scenario in the context space and estimating the delay will result in high computational complexity. Meanwhile, a set of similar contexts within a certain range often corresponds to similar delays. Therefore, we discretize each dimension of the context space and map the observed values to the partitioned grid points of the context space after observing the context information and estimating the delay based on the discretized context information (See Figure 3).

Assume that each dimension of the context space X is uniformly divided into *G* parts. Then, in total, $G^{D}$ subspaces will be included. Each subspace in X is referred to as a grid point, and the set of all grid points in X forms a context set G. Therefore, a mapping relationship from X to G can be established. Due to the fact that the value of *G* will affect the size of the context set, which, in turn, affects algorithm performance, it is necessary to set a reasonable value for parameter *G*.

The server observes the client’s contextual information before each training round and maps it to the corresponding grid points in the context set. Assuming that, before training round *t*, the context $x_{k}^{t}$ of client *k* is mapped to the grid point g∈G. If client *k* is selected to participate in this training round, the server will receive the actual delay of the client after the training is completed, denoted as τ. Subsequently, the pair (g,τ) is saved as experiences to update the corresponding estimator for the client. We denote the set of historical experiences corresponding to client *k* as $P_{k}$.

The times of client *k* participating in training before round *t* with the context falling on the context grid g∈G is recorded using a counter $C_{k}^{t}\left(g\right)$. When client *k* is selected to participate in training in round *t*, and its context falls on the context grid point *g*, the counter corresponding to the grid point *g* of client *k* will be updated, i.e., $C_{k}^{t}\left(g\right)=C_{k}^{t}\left(g\right)+1$.

In this work, the maximum likelihood estimator (MLE) is leveraged to estimate client delay. Assuming that the delay τ of client *k* corresponding to the same grid point *g* follows a normal distribution, then the estimation method can be expressed as follows:

(15) $${\hat{\tau }}_{k}^{t}\left(g\right)=\frac{\sum_{\left(g,\tau_{k}\right)\in P_{k}\left(g\right)}\tau_{k}}{C_{k}^{t}\left(g\right)}.$$

### 5.2. Exploration and Exploitation

We define the empirical threshold function as E(t), which is a monotonically increasing function of the training round *t*, representing the minimum value of $C_{k}^{t}\left(g\right)$ that the client needs to reach on any grid point if they are considered to be well explored in round *t*. Therefore, the proposed algorithm selects the subset of clients to be explored in round *t* as follows:

(16) $$E_{s,t}=\left\{k\in K_{s}|C_{k}^{t}\left(g\right)<E\left(t\right)\right\},$$

where $K_{s}$ represents the set of all clients within the cell where edge server *s* is located, and $E_{s,t}$ represents the set of clients selected to be explored. Note that if $E_{s,t}\neq \emptyset$, it indicates that there are still clients in the cell that need to be explored; thus, the cell enters the exploration phase in the current training round. Otherwise, the cell enters the exploitation phase.

(a) **Exploration Phase:** In the exploration phase, the player needs to select as many under-explored clients as possible to participate in training in order to enrich their experience and train the estimator. Here, two cases are considered, i.e., $|E_{s,t}|\;\leq N$ and $|E_{s,t}|\;>N$. In the first case, all the clients in the cell are selected to participate in this training round. After that, the player will greedily select the client with the minimum estimated delay value from the remaining clients until $|E_{s,t}|\;=N$. In the second case of $|E_{s,t}|\;>N$, *N* clients are randomly selected from $E_{s,t}$ to participate in the current round of training.

(b) **Exploitation Phase:** In the exploitation phase, the player estimates the delay of each client in the current round based on the current estimator and contextual information and selects the clients participating in the training based on the estimated values to minimize the expected delay of the current round. For each well-explored cell, the optimization problem is formulated as follows:

(17a) $$\begin{array}{cc} \min_{a(t)}\max_{k\in A_{s}\left(t\right)} & {\hat{\tau }}_{k}\left(t\right) \end{array}$$

(17b) $$\begin{array}{cc} \;\;\;\;\;\;\;\;\;s.t. & a_{k}\left(t\right)\in \left\{0,1\right\},\forall k\in K_{s}, \end{array}$$

(17c) $$\begin{array}{cc} & |E_{s,t}|=N. \end{array}$$

Problem (17) can be simply solved using a greedy algorithm, which arranges the delay of all clients in the cell in ascending order and selects the top *N* clients to participate in this training round.

## 6. Key Parameter Design

In this section, the key parameters *G* and ${\left\{E\left(t\right)\right\}}_{t=1}^{T}$ are designed to minimize cumulative delay of *T*-round training. Since the setting of parameter *G* depends on the total number *T* of training rounds, we re-express it using $G_{T}$.

### 6.1. Upper Bound of Regret

Denote the optimal solution to Problem (14) by $a^{*}=\left\{a^{*}\left(1\right),a^{*}\left(2\right),\ldots ,a^{*}\left(T\right)\right\}$. The difference between the delay corresponding to the optimal solution in each round *t* and the actual delay based on the proposed algorithm is defined as the regret, i.e.,

(18) $$r_{t}=\tau \left(t,a\left(t\right)\right)-\tau \left(t,a^{*}\left(t\right)\right).$$

The expected cumulative regret of round *T* is denoted as follows:

(19) $$E\left[R\left(T\right)\right]=\sum_{t=1}^{T}E[r_{t}]=\sum_{t=1}^{T}\left(E\left[\tau \left(t,a\left(t\right)\right)\right]-E\left[\tau \left(t,a^{*}\left(t\right)\right)\right]\right).$$

We also introduce two assumptions, as follows.

**Assumption** **1.** *For a specific estimator, as its historical experience $P_{k}$ increases, its estimation error for client delay will decrease. Therefore, it is assumed that for any grid point in the context set G, the estimator corresponding to client k satisfies the following probably appropriately correct (PAC) property:*

(20) $$\mathrm{Pr}\left\{\left|\Theta_{k}\left(P_{k}^{t}\left(g\right)\right)-\tau_{k}\left(g\right)\right|>\right\}\leq \sigma_{k}\left(\epsilon ,C_{k}^{t}\left(g\right)\right),$$

*where $\tau_{k}\left(g\right)$ represents the delay expectation when the client context x∈g is prior. $\sigma_{k}\left(\epsilon ,C_{k}^{t}\left(g\right)\right)$ is a term that decreases with the increase of the counter $C_{k}^{t}\left(g\right)$, which is related to the estimator.*

**Assumption** **2.** *Empirically, it can be inferred that when the contextual information is similar, the delay of clients is also similar. Therefore, it is assumed that for each client ∀k∈K, there exists L>0 and α>0 such that for any grid point $x,x^{\prime }\in X$ in the context space X, the following inequality holds:*

(21) $$\left|\tau_{k}\left(x\right)-\tau_{k}\left(x^{\prime }\right)\right|\leq L{∥x-x^{\prime }∥}^{\alpha },$$

*where $∥\cdot ∥$ represents the Euclidean norm in $R^{D}$.*

With the above assumptions, we have the following theorem.

**Theorem** **1.** *Given Assumptions 1 and 2, when $2H\left(t\right)+2LD^{\alpha /2}{\left(G_{T}\right)}^{\alpha }\leq At^{\theta }$, the expected cumulative regret is upper bounded as follows:*

(22) $$\begin{array}{cccc} \left[R(T)\right] & \leq r^{\max}K_{s}{\left(G_{T}\right)}^{D}\left\lceil E(T)\right\rceil \; & +\;3TLD^{\frac{\alpha }{2}}{\left(G_{T}\right)}^{-\alpha }+AT^{\theta +1} & +\;2r^{\max}\sum_{t=1}^{T}\sum_{a\in L(g^{t})}\sigma_{k^{\prime }}\left(H(t),E(t)\right). \end{array}$$

**Proof.**  Please see the Appendix A for reference. □

### 6.2. Parameter Design Based on the Upper Bound

It is assumed that the historical delay $\tau_{k}\left(x_{k}\right),x_{k}\in g_{k}^{t}$ in the experience $P_{k}\left(g_{k}^{t}\right)$ corresponding to the grid points $g_{k}^{t}∼\mathrm{CN}\left(\mu_{k}\left(g_{k}^{t}\right),\delta_{k}^{2}\left(g_{k}^{t}\right)\right)$. With the MLE, an unbiased estimate of $\mu_{k}\left(g_{k}^{t}\right)$ is given as follows:

(23) $${\hat{\tau }}_{k}\left(g_{k}^{t}\right)=\frac{1}{C_{k}^{t}\left(g_{k}^{t}\right)}\sum_{\tau \in P_{k}\left(g_{k}^{t}\right)}\tau .$$

Note that the PAC property in Assumption 1 can be further refined as follows:

(24) $$\begin{array}{ccc} \mathrm{Pr}\left\{\left|{\hat{\tau }}_{k}\left(g_{k}^{t}\right)-\tau_{k}\left(g_{k}^{t}\right)\right|>\right\} & \leq \sigma_{k}\left(\epsilon ,C_{k}^{t}\left(g_{k}^{t}\right)\right)\; & =\exp\left(-\frac{2C_{k}^{t}{\left(g_{k}^{t}\right)}^{2}}{{\left(\tau^{\max}\right)}^{2}}\right). \end{array}$$

We further let $E\left(t\right)=t^{z}\log\left(t\right)$ with 0<z<1 and $G_{T}=\left\lceil T^{\gamma }\right\rceil$ with $0<\gamma <\frac{1}{D}$. Then, the first term on the right-hand side of (22) can be rewritten as follows:

(25) $$r^{\max}K_{s}{\left\lceil T^{\gamma }\right\rceil }^{D}\left\lceil T^{z}\log\left(T\right)\right\rceil .$$

Considering ${\left\lceil T^{\gamma }\right\rceil }^{D}\leq {\left(2T^{\gamma }\right)}^{D}$, we have the following:

(26) $$\begin{array}{cccc} \; & r^{\max}K_{s}{\left\lceil T^{\gamma }\right\rceil }^{D}\left\lceil T^{z}\log\left(T\right)\right\rceil \; & \leq r^{\max}K_{s}{\left(2T^{\gamma }\right)}^{D}\left(T^{z}\log\left(T\right)+1\right)\; & =2^{D}r^{\max}K_{s}\left(T^{\gamma D}+T^{z+\gamma D}\log\left(T\right)\right). \end{array}$$

Let $2H\left(t\right)+2LD^{\frac{\alpha }{2}}{\left(G_{T}\right)}^{\alpha }\leq At^{\theta }$. Then. the third term on the right-hand side of (22) can be rewritten as follows:

(27) $$\begin{array}{cc} \; & 2r^{\max}\sum_{t=1}^{T}\sum_{a\in L(g^{t})}\sigma_{k^{\prime }}\left(H\left(t\right),E\left(t\right)\right)\\ \; & =2\left|F\right|r^{\max}\sum_{t=1}^{T}\exp\left(-\frac{2E\left(t\right)H^{2}\left(t\right)}{{\left(\tau^{\max}\right)}^{2}}\right)\\ \; & =2\left|F\right|r^{\max}\sum_{t=1}^{T}\exp\left(-2\log(t)\right)\\ \; & =2\left|F\right|r^{\max}\sum_{t=1}^{T}t^{-2}\\ \; & \leq 2\left|F\right|r^{\max}\sum_{t=1}^{\infty }t^{-2}\\ \; & \leq \frac{\pi^{2}}{3}\left|F\right|r^{\max}. \end{array}$$

Considering ${\left(G_{T}\right)}^{-\alpha }={\left\lceil T^{\gamma }\right\rceil }^{-\alpha }\leq T^{-\alpha \gamma }$, we have

(28) $$\begin{array}{ccc} E[R(T)] & \leq 2^{D}r^{\max}K_{s}\left(T^{z+\gamma D}\log\left(T\right)+T^{\gamma D}\right)\; & +\;3LD^{\frac{\alpha }{2}}T^{1-\alpha \gamma }+AT^{\theta +1}+\frac{\pi^{2}}{3}\left|F\right|r^{\max}. \end{array}$$

Let $z=\frac{2\alpha }{3\alpha +D}$, $\gamma =\frac{z}{2\alpha }$, and $\theta =-\frac{z}{2}$. Then, the highest power term of *T* in E[R(T)] is $2^{D}r^{\max}K_{s}\left(T^{\frac{2\alpha +D}{3\alpha +D}}\log\left(T\right)\right)$, where $\frac{2\alpha +D}{3\alpha +D}<1$.

Therefore, given $E\left(t\right)=t^{\frac{2\alpha }{3\alpha +D}}\log\left(t\right)$ and $G_{T}=\left\lceil T^{\frac{1}{3\alpha +D}}\right\rceil$, the expected cumulative regret increases sub-linearly with respect to *T* and the asymptotic optimal decisions of client scheduling are obtained.

## 7. Experimental Results

### 7.1. Simulation Setup

All of the experiments involved in this work are conducted on a personal computer with a CPU 2.10 GHz Intel Core i712700F and 32 GB of RAM, running a 64-bit Windows operating system and PyTorch 1.13.1. Assuming that there are 3 edge servers and 18 client devices. Each edge server can communicate with up to 2 clients simultaneously. A total of 2000 rounds of DFL training are conducted.

The communication-related parameters are configured as follows. The channel gains for both uplink and downlink links consist of small-scale and large-scale fading. The small-scale fading follows a Rayleigh distribution with uniform variance, while the large-scale fading is modeled using the path-loss equation, $\mathrm{PL}[\mathrm{dB}]=128.1+37.6\log_{10}\left(d\right)$, where *d* represents the distance in kilometers. The noise power $\sigma^{2}$ is set to −173 dBm, and the uplink resource block bandwidth is 1 Mbps. The transmit power of clients and edge servers is set to 10 mW and 1 W, respectively. The data size of model parameters is configured as $m=5\times 10^{3}$.

The local computing-related parameters for clients are configured as follows. The computing capability $s_{k}$ of each client *k* is uniformly distributed within the range $\left[10,30\right]\times 10^{6}$. The computational resource allocation $\varphi_{k}\left(t\right)$ is set to $2\times 10^{11}$. The available computational resource $\eta_{k}\left(t\right)$ follows a uniform random distribution given by $\frac{1}{1+I_{k}\left(t\right)}$ in each round, where $I_{k}\left(t\right)$ represents the number of active programs running on client *k* in round *t*, uniformly distributed within [0,10]. Furthermore, the maximum interval is set to $\tau_{\max}=5$ s. Table 1 summarizes the key parameters used in this work.

The MNIST dataset [35], comprising 70,000 grayscale images of handwritten digits (0–9), and the CIFAR-10 dataset [36], containing 60,000 color images across 10 categories, were employed for training handwritten digit recognition and image classification models, respectively. For each dataset, the data partitioning scheme was implemented as follows. After random shuffling, the dataset was uniformly distributed across all clients. In each training round, clients randomly determined the quantity of local data to utilize for model updates. For the MNIST dataset, a multi-layer perceptron (MLP) consisting of an input layer, a fully connected layer, and an output layer is chosen as the target model with a total of 101,770 trainable parameters. For the CIFAR-10 dataset, a convolutional neural network (CNN) consisting of two convolutional layers (and corresponding pooling layers), a fully connected layer, and an output layer is chosen as the target model with a total of 313,802 trainable parameters. During the training process, the batch size is set to 64. For the MNIST and CIFAR-10 datasets, the learning rates are set as 0.05 and 0.02, respectively.

To verify the effectiveness of the client selection strategy proposed in this work in reducing long-term cumulative delay, the following methods are introduced for comparison:

- Optimal client selection. In this method, the total delay of each client in each round of the system is known as a priority. When making decisions in each round, edge servers select the *N* clients with the smallest total delay in the covered cells to participate in training. Note that this method serves as the upper bound.
- ϵ-greedy client selection. This method employs a greedy metric to decide between exploration and exploitation. In the exploration round, each edge server randomly selects *N* clients from their covered cells to participate in training. In the exploitation round, each edge server selects the *N* clients with the minimum delay expectation to participate in training. This method does not utilize contextual information when making selection decisions, relying solely on randomness and delay-based selection. In this work, the value of ϵ is 0.3.
- Random client selection. At the beginning of each training round, each edge server randomly selects *N* clients from the corresponding cells to participate in the training.

### 7.2. Performance Analysis

**Figure 4:**
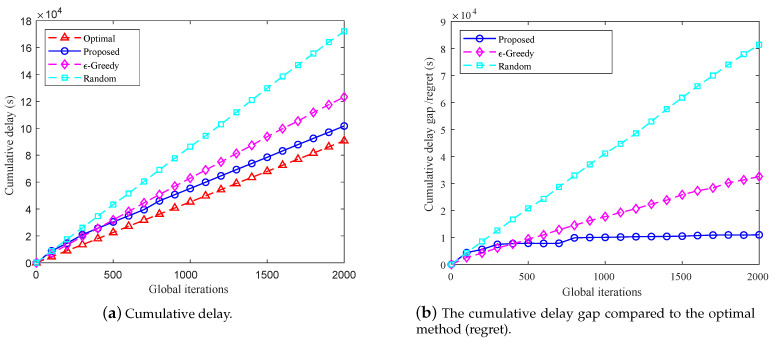
The performance comparison during the training process using different client selection methods on the MINIST dataset.

Figure 4 shows the cumulative delay and the corresponding performance regret during the training process using different client selection methods on the MINIST dataset. It can be observed from Figure 4 that, compared to the random and ϵ-greedy client selection methods, the proposed method significantly reduces delay within the given number of training rounds. This observation indicates that the proposed method can effectively mitigate the effects of heterogeneous and time-varying client resources and improve training efficiency. In addition, we find that the cumulative delay performance gap of the proposed method can gradually have sub-linear growth over communication rounds.

**Figure 5:**
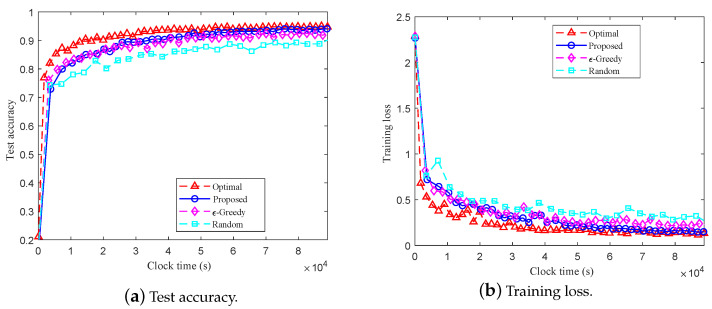
Comparison of training on the MINIST dataset using different client selection methods.

**Figure 6:**
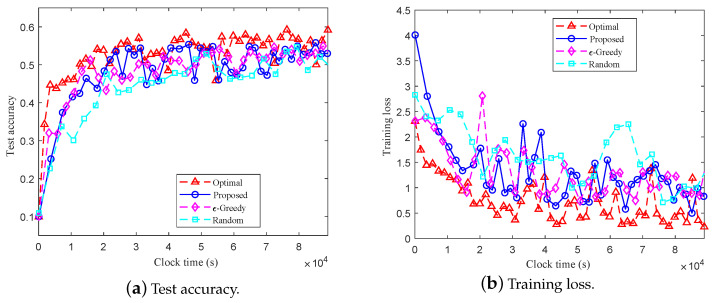
Comparison of training on the CIFAR-10 dataset using different client selection methods.

**Figure 7:**
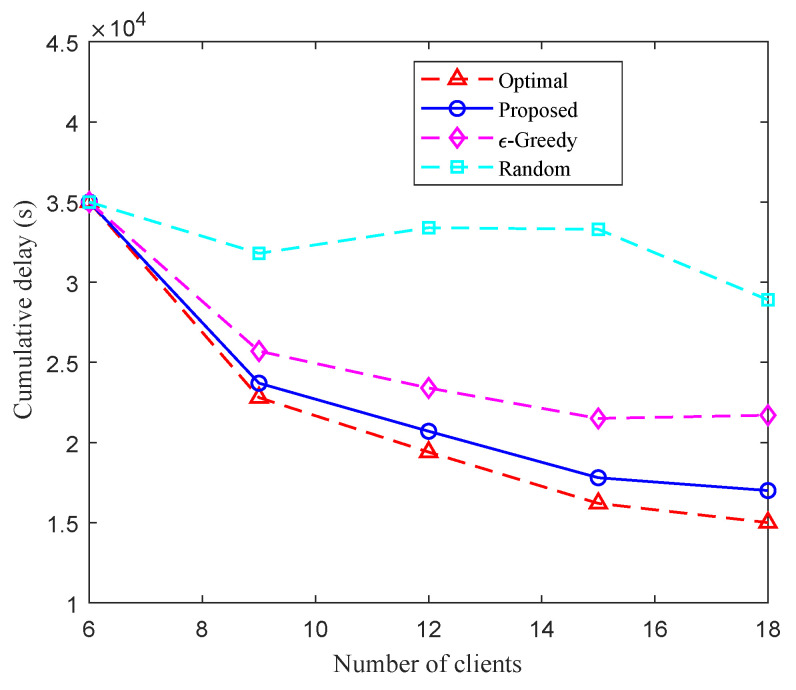
Cumulative delay versus number of clients.

Figure 5 and Figure 6 show the test accuracy and training loss over training time using different client selection methods on the MINIST and CIFAR-10 datasets, respectively. We observe that for both datasets, compared with the random and ϵ-greedy client selection methods, the proposed method can achieve the best performance, which suggests that the client selection method proposed in this work can effectively accelerate the convergence of the global model and reduce delay. Figure 7 shows the cumulative delay over different numbers of clients. As shown in Figure 7, when NS=K, i.e., all the clients can be associated with the edge BSs simultaneously, all four methods have similar delay performance. With the increase of clients (e.g., new clients joining the systems), the cumulative delay of the methods, except for the random selection method, decreases. Moreover, the cumulative delay of the proposed method is always lower than that of the ϵ-greedy and random client selection methods. The results in Figure 7 indicate that, compared to the random and ϵ-greedy client selection methods, the proposed method can effectively reduce the cumulative delay of the system.

## 8. Conclusions

This work investigates the client scheduling problem in a DFL scenario with multiple servers, where the local computing and communication resources of clients are heterogeneous and time-varying, and the aforementioned client resource priors are unknown. Firstly, model the delay generated during the FL training process and propose a client scheduling problem to minimize the cumulative delay. Subsequently, this work proposes a client scheduling algorithm based on context multi-arm slot machines. Through theoretical analysis and algorithm parameter design, this algorithm can achieve asymptotic optimal performance in theory. The experimental results show that the algorithm can make asymptotic optimal client selection decisions, and this method is superior to existing algorithms in reducing the cumulative delay of the system.

## Figures and Tables

**Table 1:** Simulation settings.

Parameters	Values
Size of the area	500 m × 500 m
Noise power spectral density	−173 dBm/Hz
Uplink resource block bandwidth	1 Mbps
Transmit power of clients	10 mW
Transmit power of servers	1 W
The data size of model parameters	$5\times 10^{3}$
The computing capability	$\left[10,30\right]\times 10^{6}$
The computational resource	$2\times 10^{11}$
The number of active programs running on client	[0,10]
The maximum interval	5 s
Batch size	64
Learning rate (MNIST / CIFAR-10)	0.05/0.02

## Data Availability

Data available on request due to restrictions. The data presented in this study are available on request from the corresponding author.

## Appendix A. Proof of Theorem 1

Due to the cumulative delay loss R(T) comprising both the exploration delay loss $R_{\mathrm{Explore}\;}\left(T\right)$ and the exploitation delay loss $R_{\mathrm{Exploit}\;}\left(T\right)$, we have $R\left(T\right)=R_{\mathrm{Explore}\;}\left(T\right)+R_{\mathrm{Exploit}\;}\left(T\right)$. Therefore, we derive the upper bounds for these two components separately to ultimately obtain an upper bound for R(T).

First, we derive the upper bound of the exploration delay loss $R_{\mathrm{Explore}}\left(T\right)$ given $G_{T}$ and E(t). If cell *s* enters the exploration phase in round *t*, there must exist some $k\in K_{s}$ and context grid $g_{k}^{t}$ such that $C_{k}^{t}\left(g_{k}^{t}\right)<E\left(t\right)$. According to the definition of E(t), there are at most ⌈E(t)⌉ exploration rounds in cell *s* in which user *k*, with context $x_{k}^{t}\in g_{k}^{t}$ and $t^{\prime }<t$, is selected to participate in training in the current round. Additionally, it can be derived that there are at most $K_{s}{\left(G_{T}\right)}^{D}\left\lceil E\left(T\right)\right\rceil$ exploration rounds across *T* rounds. Assuming the delay loss per exploration round is at most $r^{\max}$, the upper bound for the exploration delay loss $R_{\mathrm{Explote}\;}\left(T\right)$ over *T* rounds is as follows:

(A1) $$E\left[R_{\mathrm{Exploce}\;}\left(T\right)\right]\leq r^{\max}K_{s}{\left(G_{T}\right)}^{D}\left\lceil E\left(T\right)\right\rceil .$$

From this expression, it is evident that the order of $R_{\mathrm{Explore}\;}\left(T\right)$ is determined by the grid division ${\left(G_{T}\right)}^{D}$ and the function E(t).

Next, we derive the upper bound for the exploitation delay loss $R_{\mathrm{Explore}\;}\left(T\right)$ given $G_{T}$ and E(t). Let the expected delay estimate for user device *k* with context *x* be defined as $\mu_{k}\left(x\right)≜{\hat{\tau }}_{k}\left(x\right)$. Furthermore, we denote the upper and lower bounds of the expected delay estimate for user $k\in K_{s}$ across all contexts in grid *g* by ${\bar{\mu }}_{k}\left(x\right)=\sup_{x\in g}\mu_{k}\left(x\right)$ and $\mu_{k}\left(x\right)=\inf_{x\in g}\mu_{k}\left(x\right)$, respectively. We define the context at the geometric center of grid *g* as $\dot{x}\left(g\right)$ and make the following definitions:

(A2) $$\begin{array}{c} \bar{\mu }\left(g^{t}\right)=\left\{{\bar{\mu }}_{1}\left(g_{1}^{t}\right),{\bar{\mu }}_{2}\left(g_{2}^{t}\right),\ldots ,{\bar{\mu }}_{N}\left(g_{N}^{t}\right)\right\},\\ \underline{\mu }\left(g^{t}\right)=\left\{{\underline{\mu }}_{1}\left(g_{1}^{t}\right),{\underline{\mu }}_{2}\left(g_{2}^{t}\right),\ldots ,{\underline{\mu }}_{N}\left(g_{N}^{t}\right)\right\},\\ \dot{\mu }\left(g^{t}\right)=\left\{\mu_{1}\left(\dot{x}\left(g_{1}^{t}\right)\right),\mu_{2}\left(\dot{x}\left(g_{2}^{t}\right)\right),\ldots ,\mu_{N}\left(\dot{x}\left(g_{N}^{t}\right)\right)\right\}. \end{array}$$

The user selection strategy based on the delay estimate $\dot{\mu }\left(g^{t}\right)$ is defined as follows:

(A3a) $$\begin{array}{cc} \dot{a}\left(g^{t}\right)= & {\arg\min}_{a_{s}\left(t\right)}\max_{k\in A,f}\dot{\mu }\left(g^{t}\right), \end{array}$$

(A3b) $$\begin{array}{cc} \; & s.t.\;a_{k}\left(t\right)\in \left\{0,1\right\},\;\forall k\in K_{s}, \end{array}$$

(A3c) $$\begin{array}{cc} \; & \;\;\;\;∥A_{s}\left(t\right)∥=N.\; \end{array}$$

We also define the follows:

(A4) $$L\left(g^{t}\right)=\left\{a|\tau^{t}\left(a,\underline{\mu }\left(g^{t}\right)\right)-\tau^{t}\left(\dot{a}\left(g^{t}\right),\bar{\mu }\left(g^{t}\right)\right)\geq At^{\theta }\right\}.$$

We term any user selection strategy $a\in L\left(g^{t}\right)$ as a suboptimal strategy, and any strategy $a\in A,a\notin L\left(g^{t}\right)$ as a near-optimal strategy. Therefore, the exploitation delay loss can be expressed as follows:

(A5) $$E\left[R_{\mathrm{Exploit}\;}\left(T\right)\right]=E\left[R_{s}\left(T\right)\right]+E\left[R_{n}\left(T\right)\right],$$

where $E\left[R_{s}\left(T\right)\right]$ and $E\left[R_{n}\left(T\right)\right]$ represent the delay losses in the exploitation phase caused by suboptimal and near-optimal strategies, respectively. Next, we derive the upper bounds for each of these losses.

To begin with, let $W\left(t\right)=\left\{E_{s,t}=⌀\right\}$ represent the case where the algorithm enters the exploitation phase, and let $V_{a}\left(t\right)$ denote the scenario in round *t*, where the strategy is a. Consequently, the following holds:

(A6) $$\begin{array}{cc} R_{s}\left(T\right) & =\sum_{t=1}^{T}\sum_{a^{t}\in L\left(g^{t}\right)}I_{\left\{V_{a}\left(t\right),W\left(t\right)\right\}}\\ \; & \times \left(\tau^{t}\left(a^{t},\tau^{t}\right)-\tau^{t}\left(a^{*,t},\tau^{t}\right)\right). \end{array}$$

Since the maximum delay loss per round is $r^{\max}$, (A1) can be simplified to the following:

(A7) $$R_{s}\left(T\right)\leq r^{\max}\sum_{t=1}^{T}\sum_{a\in L\left(g^{t}\right)}I_{\left\{V_{a}\left(t\right),W\left(t\right)\right\}}.$$

Taking expectations on both sides of the inequality yields the following:

(A8) $$E\left[R_{s}\left(T\right)\right]\leq r^{\max}\sum_{t=1}^{T}\sum_{a\in L\left(g^{t}\right)}\mathrm{Pr}\left\{V_{a}\left(t\right),W\left(t\right)\right\}.$$

Given that in the exploitation phase with strategy a, $\tau^{t}\left(a,{\hat{\tau }}^{t}\right)\leq \tau^{t}\left(\dot{a}\left(g^{t}\right),{\hat{\tau }}^{t}\right)$, the following is true:

(A9) $$\mathrm{Pr}\left\{V_{a}\left(t\right),W\left(t\right)\right\}\leq \mathrm{Pr}\left\{\tau^{t}\left(a,{\hat{\tau }}^{t}\right)\leq \tau^{t}\left(a\left(g^{t}\right),{\hat{\tau }}^{t}\right),W\left(t\right)\right\}.$$

Defining H(t)>0, the right side of Equation (A9) includes at least one of the following events:

(A10) $$\begin{array}{cc} E_{1}= & \left\{\tau^{t}\left(a,{\hat{\tau }}^{t}\right)\leq \tau^{t}\left(a,\bar{\mu }\left(g^{t}\right)\right)-H\left(t\right),W\left(t\right)\right\},\\ E_{2}= & \left\{\tau^{t}\left(\dot{a}\left(g^{t}\right),{\hat{\tau }}^{t}\right)\geq \tau^{t}\left(\dot{a}\left(g^{t}\right),\underline{\mu }\left(g^{t}\right)\right)+H\left(t\right),W\left(t\right)\right\},\\ E_{3}= & \left\{\tau^{t}\left(a,{\hat{\tau }}^{t}\right)\leq \tau^{t}\left(\dot{a}\left(g^{t}\right),{\hat{\tau }}^{t}\right),\right.\\ \; & \left.\tau^{t}\left(a,{\hat{\tau }}^{t}\right)>\tau^{t}\left(a,\bar{\mu }\left(g^{t}\right)\right)-H\left(t\right),\right.\\ \; & \left.\tau^{t}\left(\dot{a}\left(g^{t}\right),{\hat{\tau }}^{t}\right)<\tau^{t}\left(\dot{a}\left(g^{t}\right),\underline{\mu }\left(g^{t}\right)\right)+H\left(t\right),W\left(t\right)\right\}. \end{array}$$

Thus, we have $\left\{\tau^{t}\left(a,{\hat{\tau }}^{t}\right)\leq \tau^{t}\left(\dot{a}\left(g^{t}\right),{\hat{\tau }}^{t}\right),W\left(t\right)\right\}\subseteq \left\{E_{1}\cup E_{2}\cup E_{3}\right\}$. Next, we derive the upper bound for the probability of each of these events. Given the definition ${\bar{\mu }}_{k}\left(x\right)=\sup_{x\in g}\mu_{k}\left(x\right)$, we have $E_{x\in g}\left[\mu_{k}\left(x\right)\right]=\mu_{k}\left(g\right)\leq \sup_{x\in g}\mu_{k}\left(x\right)={\bar{\mu }}_{k}\left(g\right)$, thus

(A11) $$\begin{array}{cc} \; & \;\;\;\mathrm{Pr}\left\{E_{1}\right\}\\ \; & =\mathrm{Pr}\left\{\tau^{t}\left(a,{\hat{\tau }}^{t}\right)\leq \tau^{t}\left(a,\bar{\mu }\left(g^{t}\right)\right)-H\left(t\right),W\left(t\right)\right\}\\ \; & =\mathrm{Pr}\left\{\max_{k\in K_{s}}\left(a_{k}^{t}{\hat{\tau }}_{k}^{t}\right)\leq \max_{k\in K_{s}}\left(a_{k}^{t}{\bar{\mu }}_{k}\left(g_{k}^{t}\right)\right)-H\left(t\right),W\left(t\right)\right\}\\ \; & \leq \mathrm{Pr}\left\{\max_{k\in K_{s}}\left(a_{k}^{t}{\hat{\tau }}_{k}^{t}\right)\leq \max_{k\in K_{s}}\left(a_{k}^{t}\mu_{k}\left(g_{k}^{t}\right)\right)-H\left(t\right),W\left(t\right)\right\}. \end{array}$$

The assumption (a) is as follows:

Considering that for a specific estimator, as its historical experience $P_{k}$ increases, the estimation error for user delay will decrease. Thus, we assume that the estimator corresponding to user *k* satisfies the following PAC (Probably Approximately Correct) property for any grid point in the context set G:

(A12) $$\mathrm{Pr}\left\{\left|\Theta_{k}\left(P_{k}^{t}\left(g\right)\right)-\tau_{k}\left(g\right)\right|>\epsilon \right\}\leq \sigma_{k}\left(\epsilon ,C_{k}^{t}\left(g\right)\right),$$

where $\tau_{k}\left(g\right)$ represents the expected delay for user context x∈g under prior conditions. $\sigma_{k}\left(\epsilon ,C_{k}^{t}\left(g\right)\right)$ is a term that decreases with the increase in the count $C_{k}^{t}\left(g\right)$ and is related to the estimator.

In combination with assumption (a), we obtain the following:

(A13) $$\mathrm{Pr}\left\{E_{1}\right\}\leq \sigma_{k^{\prime }}\left(H\left(t\right),C_{k^{\prime }}^{t}\left(g_{k^{\prime }}^{t}\right)\right),$$

where $k^{\prime }=\left\{k^{\prime }\in K_{s}|{\hat{\tau }}_{k^{\prime }}^{t}a_{k^{\prime }}^{t}=\max_{k\in K_{s}}\left(a_{k}^{t}{\hat{\tau }}_{k}^{t}\right)\right\}$. Similarly, we have the following:

(A14) $$\mathrm{Pr}\left\{E_{2}\right\}\leq \sigma_{k^{\prime }}\left(H\left(t\right),C_{k^{\prime }}^{t}\left(g_{k^{\prime }}^{t}\right)\right).$$

When H(t) satisfies $2H\left(t\right)+2LD^{\frac{\alpha }{2}}{\left(G_{T}\right)}^{\alpha }\leq At^{\theta }$, we have $\mathrm{Pr}\left\{E_{3}\right\}=0$. Therefore,

(A15) $$\begin{array}{cc} \mathrm{Pr}\left\{V_{a}\left(t\right),W\left(t\right)\right\} & \leq \mathrm{Pr}\left\{E_{1}\cup E_{2}\cup E_{3}\right\}\\ \; & \leq \mathrm{Pr}\left\{E_{1}\right\}+\mathrm{Pr}\left\{E_{2}\right\}+\mathrm{Pr}\left\{E_{3}\right\}\\ \; & \leq 2\sigma_{k^{\prime }}\left(H\left(t\right),C_{k^{\prime }}^{t}\left(g_{k^{\prime }}^{t}\right)\right)\\ \; & \leq 2\sigma_{k^{\prime }}\left(H\left(t\right),E\left(t\right)\right). \end{array}$$

Combined with Equation (A8), the upper bound of $E\left[R_{s}\left(T\right)\right]$ is the following:

(A16) $$\begin{array}{cc} E\left[R_{s}\left(T\right)\right] & \leq r^{\max}\sum_{t=1}^{T}\sum_{a\in L\left(g^{t}\right)}\mathrm{Pr}\left\{V_{a}\left(t\right),W\left(t\right)\right\},\\ \; & \leq 2|F|r^{\max}\sum_{t=1}^{T}\sum_{a\in L\left(g^{t}\right)}\sigma_{k^{\prime }}\left(H\left(t\right),E\left(t\right)\right), \end{array}$$

(the upper bound of $E\left[R_{n}\left(T\right)\right]$) where W(t) indicates the situation in which the algorithm enters the exploitation phase, and Q(t) denotes that the strategy $a^{t}$ in round *t* is near-optimal, i.e., $a^{t}\in A,a^{t}\notin L\left(g^{t}\right)$. So,

(A17) $$R_{n}\left(T\right)=\sum_{t=1}^{T}I_{\left\{Q(t),W(t)\right\}}\times \left(\tau^{t}\left(a^{t},\tau^{t}\right)-\tau^{t}\left(a^{*,t},\tau^{t}\right)\right).$$

Taking the expectation on both sides of the above equation, we have the following:

(A18) $$\begin{array}{cc} E\left[R_{n}\left(T\right)\right]= & \sum_{t=1}^{T}\mathrm{Pr}\left\{Q\left(t\right),W\left(t\right)\right\}\\ \cdot \; & E\left[\tau^{t}\left(a^{t},\tau^{t}\right)-\tau^{t}\left(a^{*,t},\tau^{t}\right)|Q\left(t\right),W\left(t\right)\right]\\ \leq & \sum_{t=1}^{T}E\left[\tau^{t}\left(a^{t},\tau^{t}\right)-\tau^{t}\left(a^{*,t},\tau^{t}\right)|Q\left(t\right),W\left(t\right)\right],\\ \leq & \sum_{t=1}^{T}\tau^{t}\left(a^{t},\mu^{t}\right)-\tau^{t}\left(a^{*,t},\mu^{t}\right). \end{array}$$

Since $a^{t}\in A$, $a^{t}\notin L\left(g^{t}\right)$, we obtain $\tau^{t}\left(a^{t},\underline{\mu }\left(g^{t}\right)\right)-\tau^{t}\left(\dot{a}\left(g^{t}\right),\bar{\mu }\left(g^{t}\right)\right)<At^{\theta }$, which leads to the following:

(A19) $$\begin{array}{cc} \; & \tau^{t}\left(a^{t},\mu^{t}\right)-\tau^{t}\left(a^{*,t},\mu^{t}\right)\\ \leq & 3LD^{\frac{\alpha }{2}}{\left(G_{T}\right)}^{-\alpha }+\tau^{t}\left(a^{t},\underline{\mu }\left(g^{t}\right)\right)-\tau^{t}\left(\dot{a}\left(g^{t}\right),\bar{\mu }\left(g^{t}\right)\right),\\ \leq & 3LD^{\frac{\alpha }{2}}{\left(G_{T}\right)}^{-\alpha }+At^{\theta }. \end{array}$$

Thus, the upper bound of $E\left[R_{n}\left(T\right)\right]$ is as follows:

(A20) $$\begin{array}{ccc} E\left[R_{n}\left(T\right)\right] & \leq \sum_{t=1}^{T}\left(3LD^{\frac{\alpha }{2}}{\left(G_{T}\right)}^{-\alpha }+At^{\theta }\right),\; & \leq 3TLD^{\frac{\alpha }{2}}{\left(G_{T}\right)}^{-\alpha }+AT^{\theta +1}. \end{array}$$

In summary, the upper bound of the delay loss during exploitation is as follows:

(A21) $$\begin{array}{ccc} E\left[R_{\mathrm{Exploit}\;}\left(T\right)\right] & \leq 3TLD^{\frac{\alpha }{2}}{\left(G_{T}\right)}^{-\alpha }+AT^{\theta +1}\; & \;+2r^{\max}\sum_{t=1}^{T}\sum_{a\in L\left(g^{\prime }\right)}\sigma_{k^{\prime }}\left(H\left(t\right),E\left(t\right)\right). \end{array}$$

Thus, when the condition $2H\left(t\right)+2LD^{\frac{\alpha }{2}}{\left(G_{T}\right)}^{\alpha }\leq At^{\theta }$ is satisfied, the upper bound of the total delay loss can be expressed as follows:

(A22) $$\begin{array}{cc} E[R(T)] & \leq r^{\max}K_{s}{\left(G_{T}\right)}^{D}\left\lceil E\left(T\right)\right\rceil \\ \; & +3TLD^{\frac{\alpha }{2}}{\left(G_{T}\right)}^{-\alpha }+AT^{\theta +1}\\ \; & +2r^{\max}\sum_{t=1}^{T}\sum_{a\in L\left(g^{t}\right)}\sigma_{k^{\prime }}\left(H\left(t\right),E\left(t\right)\right). \end{array}$$

The proof is complete.
